# Cancer-Associated Fibroblasts from Lung Tumors Maintain Their Immunosuppressive Abilities after High-Dose Irradiation

**DOI:** 10.3389/fonc.2015.00087

**Published:** 2015-05-12

**Authors:** Laia Gorchs, Turid Hellevik, Jack-Ansgar Bruun, Ketil-Andre Camilio, Samer Al-Saad, Tor-Brynjar Stuge, Inigo Martinez-Zubiaurre

**Affiliations:** ^1^Department of Clinical Medicine, University of Tromsø, Tromsø, Norway; ^2^Department of Oncology and Radiotherapy, University Hospital of Northen Norway, Tromsø, Norway; ^3^Department of Medical Biology, University of Tromsø, Tromsø, Norway; ^4^Department of Pathology, University Hospital of Northern Norway, Tromsø, Norway

**Keywords:** cancer-associated fibroblasts, high-dose radiation, lung cancer, tumor immunology, immunogenic cell death

## Abstract

Accumulating evidence supports the notion that high-dose (>5 Gy) radiotherapy (RT) regimens are triggering stronger pro-immunogenic effects than standard low-dose (2 Gy) regimens. However, the effects of RT on certain immunoregulatory elements in tumors remain unexplored. In this study, we have investigated the effects of high-dose radiotherapy (HD-RT) on the immunomodulating functions of cancer-associated fibroblasts (CAFs). Primary CAF cultures were established from lung cancer specimens derived from patients diagnosed for non-small cell lung cancer. Irradiated and non-irradiated CAFs were examined for immunomodulation in experiments with peripheral blood mononuclear cells from random, healthy donors. Regulation of lymphocytes behavior was checked by lymphocyte proliferation assays, lymphocyte migration assays, and T-cell cytokine production. Additionally, CAF-secreted immunoregulatory factors were studied by multiplex protein arrays, ELISAs, and by LC-MS/MS proteomics. In all functional assays, we observed a powerful immunosuppressive effect exerted by CAF-conditioned medium on activated T-cells (*p* > 0.001), and this effect was sustained after a single radiation dose of 18 Gy. Relevant immunosuppressive molecules such as prostaglandin E2, interleukin-6, and -10, or transforming growth factor-β were found in CAF-conditioned medium, but their secretion was unchanged after irradiation. Finally, immunogenic cell death responses in CAFs were studied by exploring the release of high motility group box-1 and ATP. Both alarmins remained undetectable before and after irradiation. In conclusion, CAFs play a powerful immunosuppressive effect over activated T-cells, and this effect remains unchanged after HD-RT. Importantly, CAFs do not switch on immunogenic cell death responses after exposure to HD-RT.

## Introduction

During the last decade, clinical implementation of high-dose radiotherapy (HD-RT) has revolutionized the therapeutic outcome for treatment-resistant cancers like non-small cell lung carcinoma (NSCLC). In the clinics, oligo-fractionated HD-RT regimens are often associated with stereotactic body radiotherapy (SBRT) or stereotactic ablative body radiotherapy (SABR) (1–3). Minimal margins of normal tissue in the irradiated field combined with high-precision image-guided dose-delivery and steep dose-gradients constitute the fundament for increasing dose per fraction above the conventional 2 Gy while ensuring acceptable toxicity. An understanding of the biological consequences to HD-RT in a heterotypic cellular context is still in its infancy (4), but a thorough elucidation holds the promise of optimized and refined treatment strategies in the clinics (5).

On the ground that clinical RT is affecting a well-defined volume of heterotypic tumor tissue – and questioning the dogma that direct DNA damage to neoplastic cells is the sole mechanism for tumor control mediated by RT – researchers have instigated studies of radio-responses in different tumor compartments including both epithelial tumor cells and tumor-associated stromal cells. At present, radio-responses in the diverse population of stromal immune cells are probably the best explored of the non-malignant cells (6).

From an immunological point-of-view, the clinical success of HD-RT has been crucial for revitalizing the idea of using RT as an immunological adjuvant to improve clinical outcome (7–10). Recent findings have generated enthusiasm toward the assumption that RT and immunotherapy in combination may induce so-called abscopal off-target responses which in turn could improve not only local, but also distant, tumor control (11–14) The ambitious goal of optimizing RT-induced systemic immune-responses demands scientific efforts toward fine-tuning radiation-dose and regimens (15, 16). Several reports have already indicated that HD-RT, rather than low-dose (LD)-RT, are inducing stronger pro-immunogenic responses (17–19). Interestingly, recent experiments in mammary carcinoma cells indicate that RT indeed provokes a dose-dependent enhancement of immunogenic cell death (ICD) (20). However, it is still an open question whether RT-induction of ICD in tumor cells alone is sufficient for turning a typical immunosuppressive microenvironment into a pro-immunogenic milieu capable of initiating a systemic response.

Cancer-associated fibroblasts (CAFs) are considered one of the major cellular components of the tumor microenvironment (21, 22), and high numbers of infiltrating CAFs in the tumor tissue are generally associated with poor prognosis and treatment outcome (23, 24). In contrast to normal tissue fibroblasts, CAFs are contributing to cancer progression, essentially by the secretion of a long array of soluble paracrine factors; i.e., growth factors, cytokines, and chemokines that on one hand regulate the behavior of adjacent cancer cells (25, 26) but also mediate recruitment of inflammatory and immune cells (27), as well as bone-marrow derived progenitor cells (28, 29). However, the role of CAFs as key inflammatory and sentinel cells of the immune system is less known (30–32). In fact, through cytokine and chemokine secretion, stromal fibroblasts are efficiently mediating local immunomodulation of the tumor tissue, by directly affecting trafficking, state of differentiation and activation of the broad population of immune cells. Among other observed effects, CAFs may influence T-regulatory cells functionality by production of PGE2 (33), and can impair Th1 differentiation, CTL activation, or DC maturation by production of TGF-β (34). Moreover, some recent reports highlight the indirect effects of CAFs on immune cell infiltration and function (35), consisting of regulation of angiogenesis, lymph-angiogenesis, hypoxia, extracellular matrix remodeling, and metabolism.

Ionizing radiation in a therapeutic setting affects concomitantly non-malignant as well as malignant tumor cells (36), thus provoking also tumor cell-extrinsic responses to treatment (8, 37–39). Hence, as one of the main cellular components of the tumor mass, CAFs are receiving the prescribed radiation dose in full during RT treatment, and the consequential biological effects of the treatment are dependent on radiation dose and fractionation (4). Of note, a single high radiation dose (18 Gy) is inducing permanent DNA damage responses in CAFs, accompanied with development of irreversible cellular senescence (40). Moreover, same radiation dose is able to influence the secretory profile of CAFs, thus affecting paracrine communication by CAFs (41). Herein, we shed light on the effects of radiation on the immunoregulatory properties exerted by CAFs.

## Materials and Methods

### Patients and specimens

Lung tumor specimens were obtained from seven randomly selected patients diagnosed with NSCLC and operated at the University Hospital of Northern Norway. Tumor and donor characteristics are outlined in Table 1. Average age of patients included in the study was 61 years, and none of the patients were undergoing other therapy than surgery when their samples were collected. Peripheral blood samples were obtained from healthy donors attending the University Hospital of Northern Norway Blood Bank. Samples were anonymously coded and a written informed consent was obtained from all patients and healthy volunteers in accordance with the Declaration of Helsinki. The study has been approved by the Regional Ethical Committee (REK-Nord).

**Table 1:** **Donor features corresponding to the source of CAFs used for experimentation in this study**.

Number	Age	Sex	Tumor type	T-size (mm)	T-stage and N-stage
Donor 1	60	F	MD-SCC	35	pT2aN0
Donor 2	67	F	MD-SCC	30	pT1bN0
Donor 3	62	F	BAA	12	pT1aN0
Donor 4	57	F	MD-AC	30	pT2aN1
Donor 5	64	F	LD-AC	30	pT1bN0
Donor 6	58	M	MD-AC	60	pT2bN0
Donor 7	60	F	BAA	6	pT1aN0

*BAA, bronchio-alveolar adenocarcinoma; AC, adenocarcinoma; SCC, squamous cell carcinoma; LCC, large cell carcinoma; M, male; F, female; HD, highly differentiated, MD, middle differentiated; LD, low differentiated*.

### CAF isolation, characterization and cultures

Human lung CAFs were harvested from freshly resected NSCLC tumor tissues from lung cancer patients. After surgical resection, tumor specimens were processed following a previously described protocol (40). Briefly, tumor tissues were mechanically minced into small pieces (1–1.5 mm^3^), followed by enzymatic digestion for 1.5 h at 37°C in 10 ml of Dulbecco’s modified Eagle’s medium (DMEM) containing bacterial collagenase (Cat.# C-9407 Sigma-Aldrich, St Louis, MO, USA) at a final concentration of 0.8 mg/ml. Digested tissue was spun down to eliminate collagenase, and resuspended in fresh growth medium (DMEM) supplemented with 10% fetal bovine serum (FBS) (Cat. # S0115 Biochrom, Berlin, Germany). Pure fibroblast cultures were obtained by selective cell detachment from the primary culture mix, using Enzyme-free cell detachment solution (Cat. # S-014-B EMD Millipore, Norway) and continued cell propagation in the presence of 10% FBS. Cells were grown at 3% oxygen for a short culture period (until passage two). Resulting cell cultures were characterized for purity and cell identity by flow cytometry using fluoroscein isothiocyanate (FITC)-conjugated anti-human α-smooth muscle α-actin antibody (Cat. # ab8211 Abcam, Cambridge, UK), and by immunofluorescent staining with anti-human fibroblasts activation protein (FAP) antibody (Cat. # ab53066 Abcam, Cambridge, UK) on formalin-fixed CAF-cultures (40). Immediately after determining purity, cells were harvested, counted, and cryopreserved in DMEM (Cat. # D6046 Sigma-Aldrich, St Louis, MO, USA) with 10% Dimethyl sulfoxide (DMSO) and 20% FBS at 5 × 10^5^ cells per cryotube until analysis. Before experimentation, cells were thawed in complete medium consisting of DMEM with 10% FBS and 1% penicillin-streptomycin (Cat. # P0781 Sigma-Aldrich, St Louis, MO, USA) and seeded in T-75 culture flasks.

### Preparation of CAF conditioned medium

At the third passage, CAFs were seeded at 4 × 10^5^ cells per T-75 tissue culture flasks and incubated 24 h (at 37°C) for attachment. After initial cell attachment and spreading, cultures were washed with PBS and new medium (6 ml) was replaced and cultures irradiated (1 × 18 Gy), as previously described (40). Conditioned medium (CM) from irradiated and control CAFs were collected between days 3 and 6 post-irradiation. Cell-density was 4 × 10^5^ cells/ml at the time of media collection. Supernatants were spun down by centrifugation at 2200 ×g for 5 min and then filtrated through a 0.45 μm filter for elimination of contaminant cell bodies. The resulting conditioned medium was either used immediately for experimental analysis or frozen at −80°C for further analysis.

### Isolation and culturing of human PBMCs

Peripheral blood mononuclear cells (PBMCs) from healthy volunteers were isolated using a Lymphoprep™ density gradient (Cat. #1115754 Axis-Shield, Oslo, Norway), and following standard procedures. Briefly, peripheral blood was diluted (1:1) in PBS and layered over a Lymphoprep™ gradient, followed by 30 min of centrifugation at 700 ×g at room temperature and without breaks. To remove platelets from peripheral blood, PBMCs from the resulting gradient-interface were collected using a Pasteur pipette, washed three times with 40 ml of 0.2% PBSA (0.2% bovine serum albumin in PBS), and spun down at 350 ×g for 10 min at room temperature. Purified PBMCs were counted and frozen down in 90% FBS with 10% DMSO at 5 × 10^7^ cells per cryotube. Before analysis, PBMCs were thawed in complete medium consisting of Iscove modified Dulbecco medium (IMDM) (Cat. # BE12-722F Lonza FWO-Vlaanderen, Belgium) with 10% FBS and 1% penicillin-streptomycin, and cultured in T-75 culture flasks at 37°C, in a 7.5% CO_2_ humidified atmosphere.

### Lymphocyte proliferation assays

Rates of T-cell proliferation were assessed using the carboxyfluorescein succinimidyl ester (CFSE) dilution assay. Cultured PBMCs were washed in PBS by centrifugation at 400 ×*g* for 4 min, and resuspended in 0.5 ml of PBSA (0.2%) and CFSE (Cat. # C34554 Molecular Probes, Life technologies, CA, USA) at 5 μg/ml, followed by incubation at 37°C for 15 min, and immediate cooling on wet ice and dilution in 10 ml of cold IMDM. In coculture experiments, irradiated and non-irradiated CAF cultures were established in 24-well plates, and CFSE-labeled PBMCs were added at a density of 10^6^ live cells per well. Immediately after initiation of cocultures, cells were exposed to the mitogen phytohemagglutinin (PHA) (1 μg/ml) (Cat. # 1249738 Roche, Penzberg, Upper Bavaria, Germany). Mixed cultures were incubated for 5–6 days at 37°C, with 50% medium replacement on day 3. Similar procedures were carried out for experiments with CAF-CM, but instead of cells, CAF-CM was diluted (1:1) with fresh pre-warmed lymphocyte growth medium. Following incubations, PBMCs were harvested, centrifuged at 400 ×*g* for 4 min, and resuspended in 0.5 ml PBS. CFSE fluorescence was analyzed on a FACSCalibur™(Becton Dickinson) flow cytometer, and flow cytometric data were analyzed by FlowJo (TreeStar, Ashland, OR, USA) software.

### Multiplex protein arrays (luminex)

Quantitative measurements of cytokines and inflammatory factors released into the culture medium were performed using a suspension array technique. Supernatants from CAFs, PBMCs, or cocultures were prepared as previously described, thawed and analysed using a human cytokine 10 Plex panel kit (Cat. # LHC001 Invitrogen, Life technologies, Frederick, MD, USA) according to the manufacturer’s protocol. All samples were analyzed in duplicates and in dilutions of 1:4. Levels of proteins included in the array were detected using the Bio-Plex^®^ 200 system (Bio-Rad laboratories, CA, USA), according to instructions from the manufacturer. Data were processed using Bio-Plex Manager™ 6.0 Software (BIO-RAD, CA, USA).

### Quantitative protein measurements by ELISA

Concentrations of PGE2 (Cat. # 500141 Cayman Chemical, Ann Arbor, MI, USA), TGF-β (Cat. # DY240-05 R&D Systems, Minneapolis, MN, USA), and indoleamine 2,3-dioxygenase (IDO) (Cat. # SEB547 Mu Cloud-clone corp., Houston, TX, USA) in the conditioned media from irradiated and non-irradiated CAFs, prepared as described above, were determined using ELISA kits according to the manufacturer’s instructions.

### Mass spectrometry-based proteomics

Cancer-associated fibroblasts conditioned medium from irradiated and non-irradiated cells were collected between days 3 and 6 post-treatment, concentrated in VIVASPIN centrifuge tubes (3000 MW cutoff) (Cat. # Vs0612 Startorius Stedim, Goettingen, Germany) by spinning at 2200 ×*g* for 30 min, and run in 1D SDS–PAGE to clean samples from salts and other non-protein solutes. Upon staining of gels with Coomassie Blue, the entire band spectrum was excised into three gel fractions. After excision, gel bands were subjected to in-gel reduction, alkylation, and digestion using 2–10 ng/μl trypsin (V511A; Promega, Madison, WI, USA) (41). OMIX C18 tips (Varian, Inc., Palo Alto, CA, USA) were used for sample cleanup and concentration. Peptide mixtures containing 0.1% formic acid were loaded onto a Thermo Fisher Scientific EASY-nLC1000 system and EASY-Spray column (C18, 2 μm, 100 Å, 50 μm, 15 cm). Peptides were fractionated using a 2–100% acetonitrile gradient in 0.1% formic acid over 50 min at a flow rate of 250 nl/min. Separated peptides were analyzed using a Thermo Scientific Q-Exactive mass spectrometer. Data were collected in data dependent mode using a Top10 method.

Raw data were analyzed by the MaxQuant (version 1.5.0.30) and the Proteome Discoverer 1.4 software. Fragmentation spectra were searched against the SwissProt (version 2011_12) database using an in-house Mascot server (Matrix Sciences, UK). Peptide mass tolerances used in the search were 10 ppm, and fragment mass tolerance was 0.02 Da. Peptide ions were filtered using a false discovery rate (FDR) set to 1% for peptide identifications. Protein identities in the MaxQuant software were determined using the Andromeda module, and by searching against the Uniprot Human database. Label-free protein quantification (LFQ) was also executed with the MaxQuant software. The generated list of proteins resulting from the MaxQuant software was filtered for contaminants and reverse hits in Perseus. Logarithms (log2) of the LFQ intensities were calculated, and average values for irradiated (*n* = 6) and control (*n* = 6) samples were presented with SD. Proteins appearing with less than six measurements were discarded.

### Migration assays

Chemotactic responses of activated lymphocytes were measured by a Boyden chamber assay. Briefly, PBMCs dissolved in growth medium were activated with PHA 24 h prior to starting the assay, and then 100 μl of cell suspension (containing 1 × 10^6^ cells) was added on the top chamber of Transwell culture inserts (6.5 mm diameter, 8 μm pores, Cat. # CLS3464, Sigma-Aldrich, St. Louis, MO, USA). Bottom chambers were filled with 600 μl of fresh fibroblast growth medium or with CM from irradiated and non-irradiated CAF cultures, in the presence or absence of SDF-1 (50 ng/ml) (Cat. # 300-28A PeProTech, Oak Park, CA, USA). After 2 h, all PBMCs invading the lower chamber were harvested, centrifuged at 400 ×*g* for 4 min, resuspended in 0.5 ml of PBSA, and counted with FACSCalibur™(Becton Dickinson) flow cytometer for 60 s at high flow rate. The lymphocyte population was gated on the forward and side scatter.

### ATP release assay

Extracellular release of ATP from non-irradiated (0 Gy) and irradiated CAFs (1 × 18 Gy) was measured at different time points, using the luciferin-based ENLITEN ATP assay (Cat. # FF2000 Promega, Madison, USA). Briefly, CAFs of two randomly selected donors were seeded in duplicate per time point, with density of 40,000 cells per well in a 24-well plate, and kept at 37°C. Supernatants were collected at 1, 6, and 24 h post-irradiation and centrifuged at 2200 ×*g* for 5 min. ATP-driven bioluminescence was assesed on a luminometer microplate reader (Labsystems Luminoskan). A positive control was prepared by lysing unirradiated CAFs with 0.1% Triton X-100 for 10 min.

### HMGB-1 detection by western blotting

Cancer-associated-fibroblast-supernatant HMGB-1 levels from irradiated and non-irradiated cultures, prepared as previously described, were analyzed by Western blotting at different time points. CAF-CM was collected 1, 6, 24, and 120 h after irradiation and concentrated using VIVASPIN centrifugation tubes (Cat. #Vs0612 Startorius Stedim Goettingen, Germany). Samples were then boiled in a reducing NuPAGE LDS sample buffer, resolved on NuPAGE Novex 4–12% Bis–Tris gels, and electrotransferred onto a polyvinylidene difluoride (PVDF) membrane (Millipore, Norway). Membranes were hybridized with anti-HMGB1 antibody (rabbit polyclonal to HMGB1-ChIP grade, Abcam, UK), followed by HRP-conjugated secondary antibody labeling (goat polyclonal anti-rabbit IgG, Abcam, UK). Reactions were developed by the Western Blotting Luminol reagent (sc-2048; Santa Cruz Biotechnology, USA) according to the manufacturer’s instructions. Cell lysates were collected in a Mastermix containing a 2× NuPAGE LDS Sample buffer (Invitrogen, Norway), and a 1× NuPAGE Sample Reducing Agent (Invitrogen, Norway) was used as a positive control.

### Statistical analysis

Values are expressed as means with SEM or SD as indicated. In Multiplex protein arrays and ELISAs, only readings above the detection limit of the assay are represented in figures. Data were analyzed using paired Student’s *t*-test, and *p*-values <0.05 denoted the presence of statistically significant differences. All statistics were determined with Graph pad prism version 5.00 for Windows (Graphpad).

## Results

**Figure 1:**
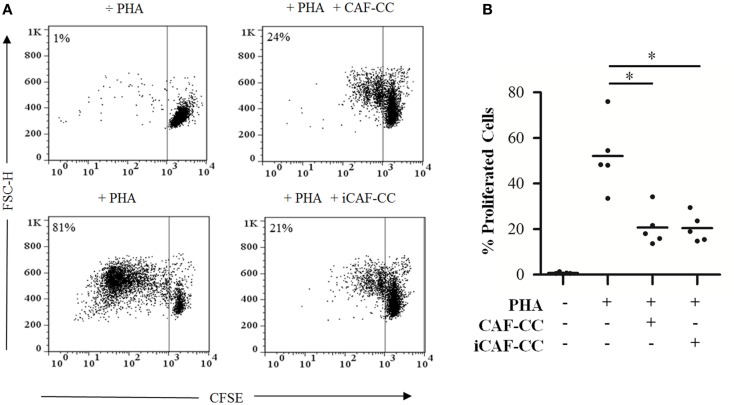
**T-cell proliferation assays: immunosuppressive effects elicited by irradiated and control CAFs during coculture**. T-cell proliferation assays were performed using CellTrace™ CFSE labeled human PBMCs activated with 1 μg/ml PHA. Cells were cocultured with irradiated (iCAF-CC) or non-irradiated CAFs (CAF-CC) at a ratio of 1:100 CAF:PBMC and allowed to proliferate for 5 days. Assays were performed with CAFs isolated from five randomly selected donors. T-cell proliferation was determined by measuring CFSE fluorescence intensity by flow cytometry after gating the lymphocyte population by forward and side scatter. **(A)** Representative flow cytometry dot plots showing percentage of CFSE^low^-labeled T-cells. One out of five representative experiments is shown. **(B)** Graph shows rate of T-cell proliferation determined by loss of CFSE fluorescence. Data points are representative for five different CAFs donors with bars representing means from triplicate experiments. Student’s *t*-test value (*p < 0.05).

Prominent immunosuppressive abilities from CAFs have been suggested in previous studies performed in immunocompetent animal models (32). Based on this premise, we initially checked whether we could reproduce such effects in functional assays *in vitro*, and whether HD-RT affects in some way the potential immunoregulatory functions of CAFs. To this end, we performed T-cell proliferation assays comprising PHA-activated lymphocytes in coculture with irradiated (iCAFs) or non-irradiated CAFs (Figure 1). Indeed, CAFs exerted a potent immunosuppressive effect on proliferating T-cells in a donor-independent manner, and this effect was similar between irradiated and non-irradiated CAFs.

**Figure 2:**
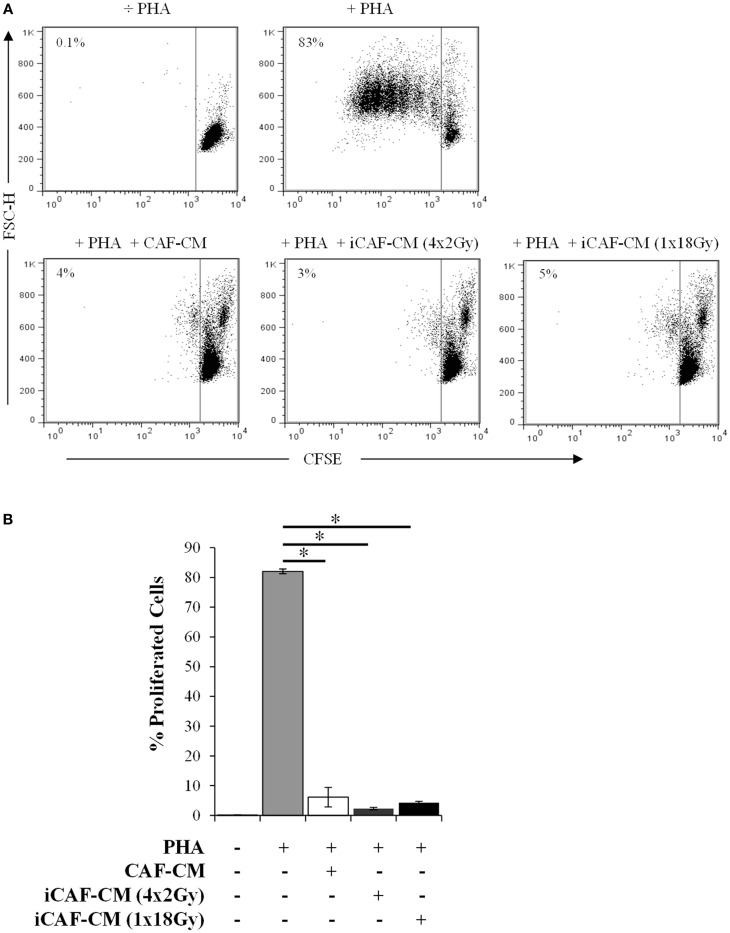
**T-cell proliferation assays: immunosuppressive effects elicited by irradiated and control CAF-CM**. T-cell proliferation assays were performed using CellTrace™ CFSE labeled human PBMCs activated with 1 μg/ml PHA and cultured with CAF-CM at a 1:1 ratio for 5 days. Assays were performed with CM harvested 3 days post-RT from irradiated (iCAF-CM) or non-irradiated (CAF-CM) CAFs cultures. T-cell proliferation was determined by measuring the CFSE fluorescence intensity by flow cytometry after gating the lymphocytes population by forward and side scatter. **(A)** Representative flow cytometry dot plots showing the percentage of CFSE^low^-labeled T-cells. One out of three representative determinations is shown. **(B)** Graph shows the rate of T-cell proliferation determined by CFSE fluorescence loss. Data points are representative for three different CAF donors with bars representing means from triplicate determinations. Student’s *t*-test value (**p < 0.001).

Several reports have suggested that the immunoregulatory properties of CAFs are mediated via the release of soluble factors that directly or indirectly affect lymphocyte activation and/or recruitment (35, 42). We thus explored if the same immunosuppressive effects observed during CAFs-lymphocytes cocultures could be reproduced by using only the CM (Figure 2). Results showed that the inhibitory effect was indeed mediated via paracrine signaling, since similar effects could be reproduced by CAF-CM, and almost identical outcomes were observed with both irradiated and non-irradiated cells. Of importance, in these experiments we were checking also whether low-dose fractionated radiation had same or different outcomes as compared with high-dose radiation. In fact, immunosuppressive effects remained unperturbed under both radiation regimens.

**Figure 3:**
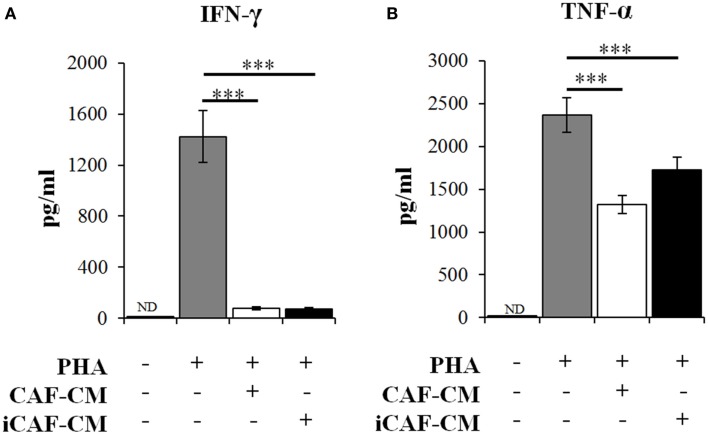
**Inhibition of T-cell derived immunogenic cytokines secretion by conditioned medium from irradiated and control CAFs**. Quantitative levels of **(A)** IFN-γ and **(B)** TNF-α were measured by multiplex cytokine immune-assays in the supernatants of T-cell cultures. Experimental groups include PBMCs in regular growth medium or PBMCs in CM obtained from irradiated (iCAF-CM) and non-irradiated (CAF-CM) CAF cultures. Data represent mean ± SD of five different CAF donors from triplicate experiments. Student’s *t*-test value (***p < 0.0001). ND; non-detected.

To further explore the immunosuppressive functions of CAFs and iCAFs, we checked the expression of effector cytokines by activated T-cells exposed to CAF-CM (Figure 3). In line with our initial observations, culture supernatants of both irradiated and non-irradiated CAFs were able to efficiently inhibit secretion of the immunoregulators IFN-γ and TNF-α in a similar way. Of note, neither IFN-γ nor TNF-α could be detected in supernatants of CAFs or iCAFs.

**Figure 4:**
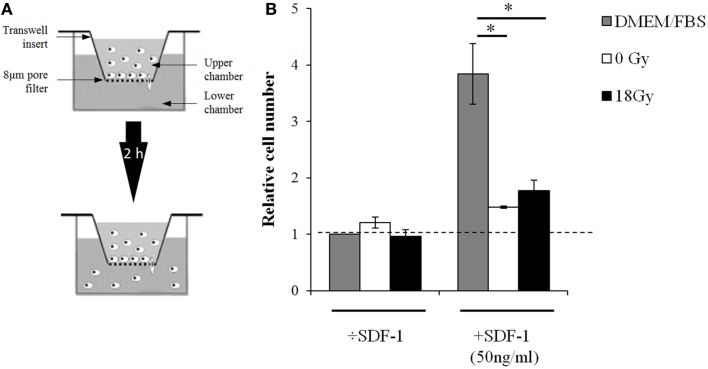
**CAF-CM from irradiated and non-irradiated cultures inhibits the migratory capacity of activated lymphocytes**. **(A)** Experimental setup for lymphocyte migration assays using polycarbonate filter chambers with 8 μm pore size. PBMCs (10^6^), activated 24 h prior to assaying, were added to the upper chambers while the lower chambers contained growth medium or conditioned medium with or without SDF-1. The assay was run for 2 h at 37°C and cells migrating to the lower chambers were quantified using flow cytometry with appropriate lymphocyte gating. **(B)** Relative migration rates, corresponding to percentages of migrating cells expressed in relation to untreated group. Each bar represents mean value (±SD) of two different CAF donors, each measured in triplicates. Student’s *t*-test value (*p < 0.05).

To our knowledge, CAF-mediated effects on lymphocyte migratory functions have not been studied previously. Given the striking suppressive actions achieved with CAF-CM on T-cell activation, we checked whether supernatants from CAFs and iCAFs could influence the migratory abilities of lymphocytes in Transwell insert systems (Figure 4). The effects were checked in the presence or absence of the potent leukocyte chemoattractant stromal-derived factor 1 (SDF-1). In line with the preceding findings, supernatants from CAFs and iCAFs could equally block migration of lymphocytes exposed to SDF-1. Of importance, this effect could only be reproduced if the lymphocytes were pre-activated with PHA before the migration assay.

**Figure 5:**
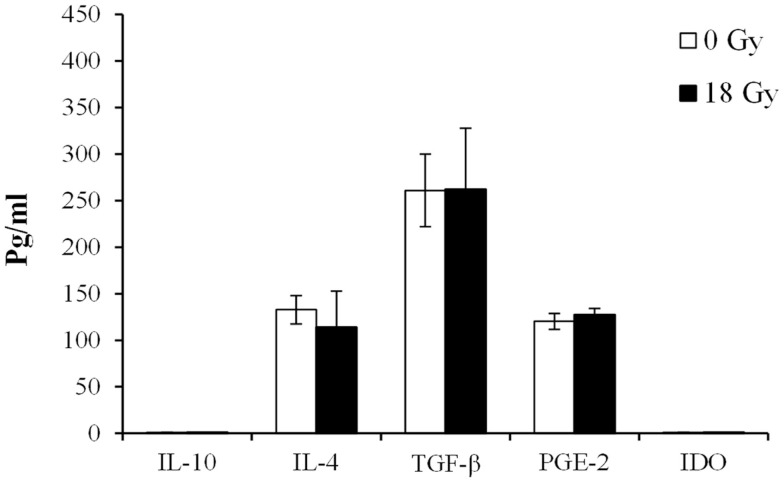
**Levels of immunosuppressive factors expressed in supernatants of irradiated and non-irradiated CAF cultures**. Levels of immunosuppressive factors were measured by ELISA in conditioned medium obtained from irradiated (18 Gy) or non-irradiated (0 Gy) CAF cultures. Each bar represents mean ± SD of five different CAF donors from triplicate experiments. ND, non-detected.

To deepen our understanding on the potential CAF-derived soluble mediators behind the observed effects, we quantified the expression of acknowledged immunosuppressive molecules in the culture medium from CAFs and iCAFs by immunobased assays (Figure 5). Anti-inflammatory molecules such as interleukin (IL)-10 or IDO were not detected in CAF supernatants; however, other important immunoregulators such as IL-4, TGF-β, or PGE2 were detected in the culture medium, but at relatively low levels. The results show approximately same amounts of all three factors in supernatants from both irradiated and non-irradiated cells.

**Figure 6:**
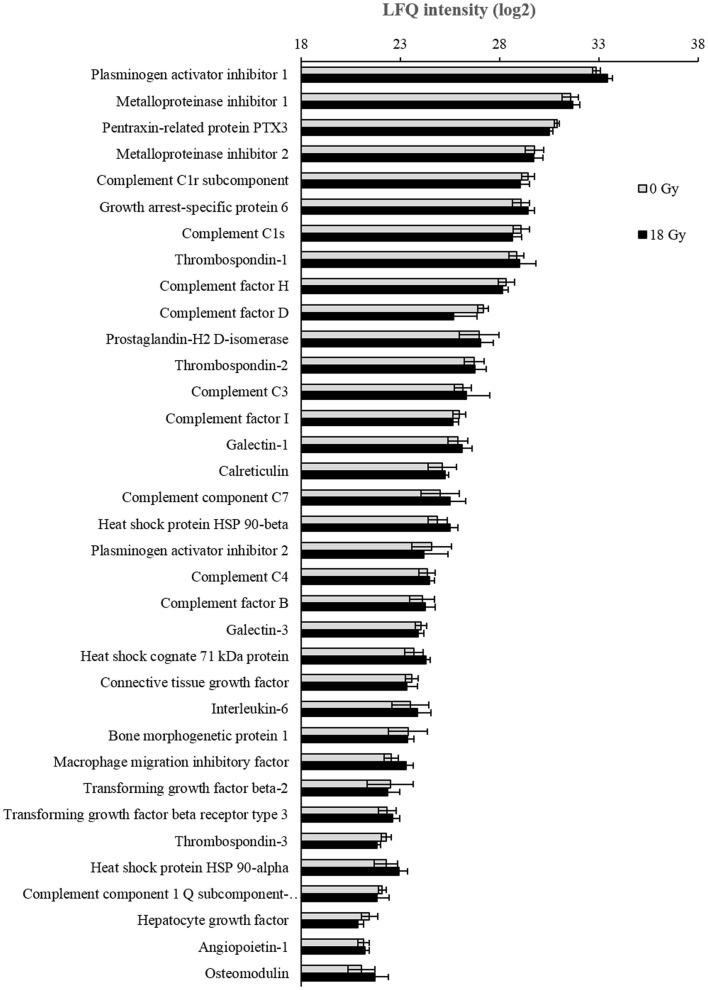
**Analyses of CAF secretome by mass spectrometry**. Conditioned medium from CAFs and iCAFs concentrated by ultrafiltration was analyzed for total protein content by LC-MS/MS proteomics. In this figure, only proteins relevant in the context of immunoregulation and expressed by most CAF donors are represented. Logarithms (log2) of Label free quantification (LFQ) intensities were calculated, and average values for irradiated (*n* = 6) and control (*n* = 6) samples were presented with standard deviations.

Our study of CAF-derived soluble signals with immunoregulatory potential was then broadened by applying LC-MS/MS proteomics on concentrated cell culture supernatants. Protein identification and classification was achieved through computational analysis, including bioinformatics and systems biological interpretation of results. Biological pathways and protein functions including cell communication, immunosystem processing, and secretion were annotated by Gene Ontology Biological Process in Perseus. Proteomic analyses of the secretome from the six donors gave a total yield of 978 identified proteins, of which 261 had relevant inflammatory or immunomodulatory functions. Full list of identified proteins from each donor is included in the Table S1 in Supplementary Material. From the last mentioned group, a selection of 35 immunoregulatory proteins, which could be quantified in supernatants from at least two of the donors, was further used for comparative analyses. LFQ intensities of each protein were calculated by the MaxQant software, and average expression values from all six donors were determined. As shown in Figure 6; no significant changes were observed between controls and irradiated cells in any of the 35 selected proteins.

**Figure 7:**
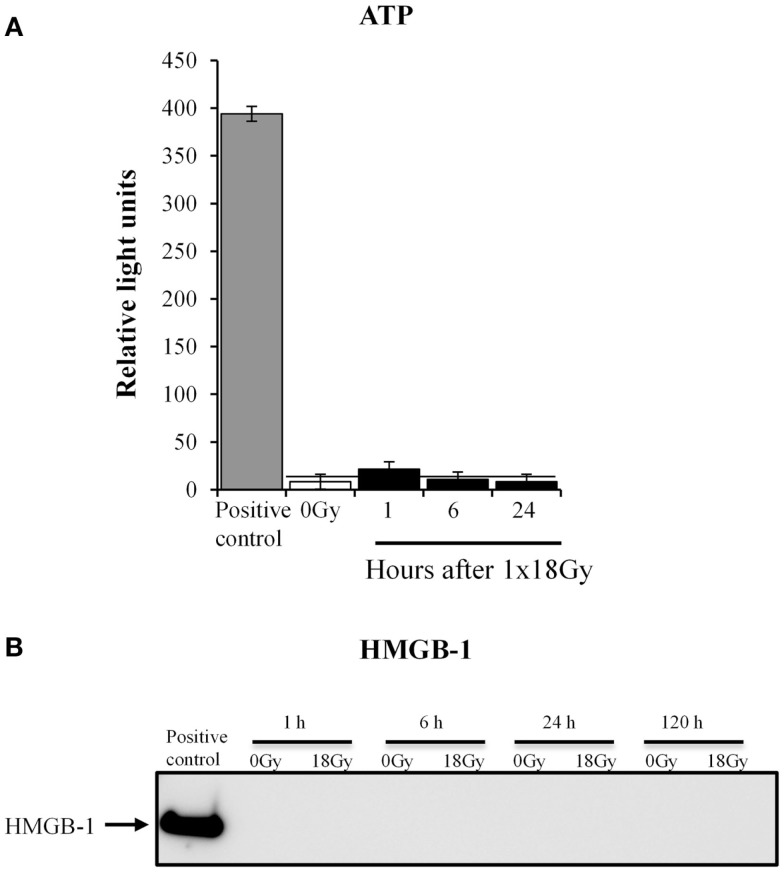
**Irradiated CAFs do not release immunogenic cell death signals**. **(A)** Relative levels of extracellular ATP in irradiated (1 × 18 Gy) CAF cultures were measured at 1, 6, and 24 h post-irradiation. Results were compared with levels of ATP released by non-irradiated cells (0 Gy) and with a positive control (CTR+) obtained after cell lysis. There were no significant differences between ATP released by irradiated and non-irradiated cells at any time point. Bars represent mean value ± SD from two different CAF donors. Horizontal bar is shown for comparison of values. **(B)** Expression of HMGB1 in CAF supernatants was analyzed from cells representing 1, 6, 24, and 120 h after irradiation (18 Gy) by Western blot, and compared with levels of HMGB1 released by non-irradiated cells at the indicated time point. Results show no detectable release of HMGB1. Data are from one representative experiment out of two independent experiments. Intracellular HMGB1 measured in whole cell lysates was used as a positive control.

HD-RT has been shown to enhance ICD responses in different types of cancers (20). Given the increasing attention put on ICD responses exerted by radiotherapy (43), we aimed at addressing whether also CAFs turn on ICD mechanisms after exposure to high-dose irradiation. ICD is characterized by the release of danger signals or alarmins from damaged or dying cells, including molecules such as ATP and high motility group box-1 (HMGB-1) (44). We thus explored the presence of ATP and HMGB-1 in the culture medium from CAFs collected at different time points post-irradiation (1 × 18 Gy) (Figure 7). Our results clearly demonstrate that CAFs do not turn on ICD mechanism after exposure to HD-RT, as neither ATP nor HMGB-1 could be detected in the conditioned medium.

## Discussion

High-dose radiotherapy is increasingly being used in the clinics for treatment of solid malignancies, and this approach is associated not only with higher rates of loco-regional tumor control but also with the induction of secondary effects which influence therapy outcomes, such as changes in the vasculature and the immune status of tumors (4). However, the effects of radiation in key non-malignant cellular components of tumors have not received much attention. In this study, we aimed at uncovering the responses of tumor fibroblasts (CAFs) to HD-RT in relation to their function as regulators of tumor immunity. Three main observations can be highlighted from our study: (i) CAFs display a powerful immunosuppressive effect over T-cells, affecting both their function and migration rates; (ii) Neither HD-RT (1 × 18 Gy) nor LD-RT (4 × 2 Gy) is influencing the immunosuppressive functions of CAFs *in vitro*; and (iii) HD-RT do not ignite ICD responses in CAFs.

Ionizing radiation modifies the tumor cell phenotype and induces a tumor microenvironment that fosters innate and adaptive immune responses. At high doses, RT enhances tumor cell injury and the release of proteins from inside the cells that may alert the immune system. Concomitantly, HD-RT has been associated with an increased surface expression of molecules by tumor cells with immuno-modulating functions such as MHC class I (45), ICAM-1, and the death receptor CD95 (46). On the other hand, the activation status of inflammatory cells may also be altered by radiation. Previous reports have suggested that HD-RT favors the presence of type-2 macrophages with tolerogenic functions, an effect associated with induction of immune inhibitory molecules such as COX-2/PGE-2 (47). Whereas low-dose irradiation may redirect macrophage differentiation from the immunosuppressive state (type-2) to one that enables recruitment of cytotoxic T-cells. Also, LD-RT has been shown to reduce the production of reactive oxygen species and the activity of inducible nitric oxide synthase by activated macrophages (48). Despite the accumulating literature on the immunomodulatory effects exerted by radiotherapy on the different cellular components in malignancies, it remains unexplored how CAFs respond to radiotherapy in regard to their important immunoregulatory roles. In this work, we disclose that neither HD-RT, given as a single fraction of 18 Gy (clinically relevant dose in the context of SABR), nor LD-RT, given as fractionated small doses (clinically relevant in the context of conventional RT), is altering the immunoinhibitory functions of CAFs.

The role of CAFs as immunoregulatory cells in tumors has been studied to some extent in the past; however a general consensus on the ultimate role played by these cells on local immunoreactions is still lacking. Animal studies, like the ones by Kraman et al. (32) or Liao et al. (49), highlighted already some years ago the important role played by CAFs as crucial immunosuppressive cells, emphasizing the capacity of CAFs to influence immune polarization toward a permissive, tumor-promoting microenvironment (32, 49). However, this view has later on been challenged by other studies, which demonstrate CAF-mediated anti-tumor immune responses in pancreas cancer (50), or a bidirectional role observed with CAFs from non-small cell lung cancer in coculture experiments with tumor-associated T-cells (42). To ascertain the role played by CAFs as immunoregulators in our system, we initially ran T-cell proliferation assays in coculture conditions with CAFs. Our results showed a very clear and reproducible inhibitory effect exerted by CAFs. Of note, this effect was basically identical for both non-irradiated and irradiated CAFs, and independent on the radiation dose and fractionation schedules applied. Additionally, we observed that the effect is primarily mediated via release of paracrine signals, since very similar results were reproduced by only using the CM. Our *in vitro* functional studies also included effector cytokine expression by T-cell assays and migration assays. Both sets of experiments reinforced the initial observation on CAF-mediated immunosuppressive actions, and bring out the fact that CAFs are able to down-regulate both the activation status and the migration (or recruitment) of PHA-activated T-cells. Of importance, in all functional assays, radiation exposure of cells did not contribute to variations on the observed effects.

Through the production of miscellaneous soluble signals including chemokines, cytokines, and extracellular matrix molecules, CAFs are able to influence the trafficking and activation status of T-lymphocytes. Acknowledged immunosuppressive molecules produced by CAFs include TGF-β1 (34), PGE2, IDO (33), or tenascin-C. All these molecules exert a direct negative effect on the acquisition and the expression of T-cell effector functions. Additionally, some of these factors are also known to deactivate or suppress NK cells. Moreover, CAFs may contribute to the accumulation and/or differentiation of regulatory T-cells in tumors through the expression of TGF-β (51) or the chemokine CCL5/RANTES (52). In our study, we have checked the expression of some of the mentioned proteins as well as other recognized immunosuppressive molecules by quantitative approaches. Signaling molecules such as IL-4, TGF-β, and PGE2 could be readily detected in CAF cell culture supernatants; however, their expression remained unchanged after exposure to a radiation-dose of 18 Gy. Additionally, the CAF secretome was analyzed by a proteomic approach. Mass spectometry data put forward a list of more than 260 proteins released by CAFs with immunomodulatory functions. Interestingly, we identified several complement components and also well-known inflammatory regulators such as IL-6, prostaglandin H2, and macrophage colony-stimulating factor (CSF-1). Of note, immunomodulators such as pentraxin and galectin-3 could be identified in the culture media of both CAFs and iCAFs. These molecules represent interesting leads for further investigation of the mechanism behind the CAF-mediated immunoregulatory actions. The overall findings on soluble signal molecules are in agreement with our observations in the functional assays no differences were found on immunosuppressive functions between irradiated and non-irradiated cells. However, it is still undefined which CAF-derived factor, or combination of factors, is ultimately responsible for the inhibitory effect observed on T-lymphocytes.

HD-RT is associated with increased tumor-antigen expression and the induction of necrotic forms of tumor cell death. Consequently, intracellular proteins naturally occurring in the nucleus, organelles, or the cytoplasm are released. Such molecules operate as damage-associated molecular patterns (DAMPs) or alarmins (53), and have the potential to prime the immune system and mount an immune attack. This phenomenon is now defined as ICD (20, 54). Well-studied danger signals include release of the DNA binding protein HMGB-1, cytosolic ATP, the stress factor heat-shock protein 70, and the plasma membrane translocation of endoplasmic reticulum-associated calreticulin. Again, existing literature on ICD-responses in the context of radiation have essentially considered responses from dying tumor cells; however, no attention has been paid to the contribution of CAFs in this matter. Here, we report for the first time that HD-RT does not trigger ICD in human CAFs, as no release of ATP or HMGB-1 could be measured in CAF-CM at any time point.

Still, much of the complex interactions between stromal fibroblasts and the different arms of the immune system in cancers remain unknown. By using fresh human material, we show here that CAFs from NSCLC exert powerful immunosuppressive effects on T-lymphocytes. In our laboratory, we are currently making efforts to track down the CAF-derived soluble factors responsible for this effect. Of importance, HD-RT, which is able to induce cellular senescence and other important phenotypic changes on CAFs, do not affect the immunoregulatory actions of these cells. Moreover, HD-RT, which is known to induce ICD-responses in dying tumor cells, does not trigger ICD in CAFs. We present here important new clues that help us understand the overall responses of tumor constituents to radiotherapy, and we believe that our data should be taken into account at the time of considering HD-RT as an immunoadjuvant in clinical settings.

## Author Contributions

LG has been implicated in most experimental work, conducting all functional assays, and protein determination assays. TH has established and conducted protocols for irradiating cells and helped drafting the manuscript. JB has been involved in proteomic part of the study, including data collection, and interpretation. KC has conducted experiments regarding immunogenic cell death and has helped in drafting the manuscript. SA has been in charge of collecting tissue specimens and gathering clinical data from donors. TS has a substantial contribution in the conception and designing of the work and has been implicated in interpretation of results. IM-Z has been the main coordinator of the study, had a main role in conception and design of the work, and has been involved in drafting the manuscript.

## Conflict of Interest Statement

The authors declare that the research has been conducted in the absence of any commercial or financial relationships that could be construed as a potential conflict of interest.

## Supplementary Material

The Supplementary Material for this article can be found online at http://www.frontiersin.org/Journal/10.3389/fonc.2015.00087/abstract
